# Acetone-Sensitive Thin Films Comprising Coal Fly Ash Na-X Zeolites and Sol–Gel Nb_2_O_5_ Matrix †

**DOI:** 10.3390/nano11092399

**Published:** 2021-09-15

**Authors:** Katerina Lazarova, Silviya Boycheva, Marina Vasileva, Denitza Zgureva-Filipova, Biliana Georgieva, Tsvetanka Babeva

**Affiliations:** 1 Institute of Optical Materials and Technologies “Acad. J. Malinowski”, Bulgarian Academy of Sciences, Akad. G. Bonchev Str., bl. 109, 1113 Sofia, Bulgaria; marina@iomt.bas.bg (M.V.); biliana@iomt.bas.bg (B.G.); 2 Department of Thermal and Nuclear Power Engineering, Technical University of Sofia, 8 St. Kl. Ohridsky Blvd., 1000 Sofia, Bulgaria; sboycheva@tu-sofia.bg; 3 College of Energy and Electronics, Technical University of Sofia, 8 St. Kl. Ohridsky Blvd., 1000 Sofia, Bulgaria; dzgureva@gmail.com

**Keywords:** acetone, sensing, optical sensor, coal fly ash zeolites, thin films, Nb_2_O_5_

## Abstract

In this study, thin composite films of a sol–gel Nb_2_O_5_ matrix doped with coal fly ash Na-X zeolites were deposited by the spin-coating method. Fly ash of lignite coal collected from the electrostatic precipitators of one of the biggest TPPs in Bulgaria was used as a raw material for obtaining zeolites. Zeolite Na-X was synthesized by ultrasonic-assisted double stage fusion-hydrothermal alkaline conversion of coal fly ash. In order to improve the optical quality and sensing properties of the deposited thin films, synthesized zeolites were wet-milled for 60, 120, and 540 s prior to film deposition. The surface morphology of zeolite powders was studied both by scanning electron microscopy and transmission electron microscopy, while their porosity was investigated by N_2_-physisorption. Refractive index, extinction coefficient, and thickness of the films were determined through fitting of their reflectance spectra. The sensing ability of thin films towards acetone vapors was tested by measuring the reflectance spectra prior to and during exposure to the analyte, and the change in the reflection coefficient ∆R of the films was calculated. The influence of milling time of zeolites on the sensing and optical properties of the films was assumed and confirmed.

## 1. Introduction

With their excellent chemical and thermal stability, zeolites can be used in the preparation of compounds and devices with desirable fundamental physical and chemical properties. A wide variety of applications of zeolite materials in gas sensors have already been developed depending on their characteristics [1]. For example, optical humidity sensing of zeolites is achieved by inserting methylene blue molecules into the pores of their framework structure or by assembling host-guest zeolite composites with substances exhibiting excellent hygroscopic ability, such as lithium chloride [2,3]. Gas sensor systems responsive via resonance frequency shifts of microbalance oscillators have been assembled by deposition of zeolites onto quartz crystal [4,5]. Novel generation impedance spectroscopy type sensing devices have been proposed based on the direct interaction of gas molecules with the self-organized extra-framework cations and complexes stipulated by nanoconfinement effects into the pores, thus resulting in altering the conductivity of the zeolites [6,7]. Zeolites integrated into gas sensor devices as physical or chemical filter materials enhance their selectivity and sensitivity to specific molecules, providing reliable performance and ultra-low detection limits [8,9]. Metal oxide-doped zeolites or zeolite/metal oxide layered structures have shown a strong response and selectivity as active media in semiconducting sensors [10,11,12]. Adding zeolites to a metal oxide matrix is a way to increase the overall porosity when deposited in a thin film form [13]. In addition to the intrinsic microporosity of the zeolites, additional free volume (air) into the samples could be introduced and managed by varying the volume fractions depending on the concentration and size of the zeolite particles [14].

Among all known zeolites, there are uncommon ones, and an example of rare natural zeolites is the Faujasite (FAU). In practice, the synthetic analogue of FAU in sodium form Na-X is widely used as an adsorbent, dryer, molecular sieve, catalytic carries, host matrix, and so on, because of its structural supercage, large pore size, and high specific surface area [15]. Zeolite Na-X is synthesized relatively easily at mild conditions from reaction mixtures with a high SiO_2_/Al_2_O_3_ ratio and using sodium hydroxide as an alkaline activator [16]. In the context of the circular economy for environmentally friendly waste management and saving of raw materials, the production of Na-X zeolite is intensively studied by utilization of waste aluminosilicates. It can also be obtained by processing of fly ash (FA) from combustion of coal, which is generated in enormous amounts as a by-product in the energy production from coal-fired thermal power plants (TPPs). Typically, FA contains above 70 wt. % of amorphous and crystalline aluminosilicates [17]. The conversion of FA to zeolites is based on the alkaline dissolution of the aluminosilicates, hydrogel formation, and its heterogeneous crystallization into the determined zeolite phase by different synthesis approaches. The most applied conversion techniques are atmospheric crystallization, hydrothermal activation, and double-stage fusion-hydrothermal synthesis. Zeolite Na-X crystallizes as a metastable phase and can be obtained at mild conditions (temperatures and alkalinity) by the all three synthesis approaches. An energy-saving process through continuous alkaline transformation of FA at ambient temperatures also results in zeolite Na-X [18]. However, the highest degree of conversion of raw FA to a single Na-X phase is achieved by applying two-stage synthesis with a preliminary fusion stage of the reaction mixture and subsequent hydrothermal activation [19,20]. The pre-fusion of the raw FA with alkaline activator prior to the hydrothermal treatment contributes to better utilization of the raw material by supporting the solubility of the resistant crystalline phases in the FA composition (quartz, mullite, and anorthite) and their conversion into the final zeolite product. The one-step hydrothermal process can also provide zeolite Na-X, but commonly accompanying phases also crystallize [21]. The homogenization of the reaction mixtures by ultrasonic treatment results in nanocrystalline morphology of the obtained zeolite Na-X and uniform distribution into the zeolite matrix of the transition metal oxides transferred from the raw FA [19]. In addition, the sonication ensures a high surface-to-volume ratio and large external surface of the zeolite particles owing to the finer fragmentation of the crystallization seeds caused by the ultrasonic cavitation effect. The developed external surface, the nanocrystalline particle size, and the uniformly distributed metal oxides into the zeolite matrix are essential for sensor material applications.

As mentioned above, the purpose of using zeolites in sensors is usually to improve sensitivity and selectivity. When used in thin films, this is achieved via preferential adsorption and in order to assure a fast response time of the sensing element. It is difficult to also attain a balance between the choice of a suitable substrate, a material that, when deposited with spin coating, forms thin films of good optical and mechanical quality and, of course, the layer to be sensitive to a particular analyte. Among all types of sensing methods, the optical sensing via thin analyte-sensitive film, which detects alteration in the environment, such as the presence of volatile organic compounds (VOCs), via color/reflectance/transmittance change, is particularly interesting [22,23,24]. The reasons behind this are the following facts: (i) there is no need for an electrical power supply and a specially trained specialist to operate the optical sensor because its working principle presupposes an intuitive detection method, especially in the case when color change is used for detection; (ii) optical sensors work at room temperature, making them very suitable for flammable and explosive environments; and (iii) they have relatively low cost thanks to the simple preparation and detection methods. The summary of the latest developments in the application of zeolites as sensor media and filters reveals the still unexplored potential related to the functionalization of these materials [6]. Coal fly ash zeolites are relatively new materials and the opportunities for their application are still being studied. These materials combine the advantages of zeolites with some unique features, e.g., they are characterized by mixed micro-mesoporosity and contain extra-framework ions and metal oxides transferred from the raw ash. These features are prerequisites for pronounced confinement effects and surface reactivity, which are essential for the sensor materials. At present, the studies on the optical and sensing properties of coal ash zeolites are quite superficial and, in this sense, this research is of great novelty.

In this paper, we use niobium sol and Na-X zeolites synthesized from coal fly ash by ultrasonic-assisted double-stage fusion-hydrothermal alkaline conversion to deposit thin composite films via the spin coating method. The films consist of Nb_2_O_5_ matrix with FA zeolites embedded inside. In order to evaluate the effect of zeolites’ size on optical and sensing properties of the composite thin films and achieve different levels of porosity, zeolites were wet-milled for 60, 120, and 540 s prior to the incorporation in the sol–gel matrix. Optical and sensing properties toward acetone were studied through reflectance measurements before and during exposure to testing vapors. The utilization of Nb_2_O_5_/Na-X zeolites thin films as sensitive elements for optical sensing of acetone is demonstrated and discussed.

## 2. Materials and Methods

FA from the electrostatic precipitators of the lignite coal supplied TPP “AES Galabovo” in Bulgaria was collected in order to be utilized for the synthesis of Na-X zeolite. Powder samples were synthesized via ultrasonic-assisted double-stage fusion-hydrothermal alkaline conversion and were studied with respect to their phase composition, morphology, and surface properties [25]. The solid mixture of sodium hydroxide and FA in a ratio of 2:1 was pre-fused at 550 °C for one hour. The obtained sintered product was disintegrated and dispersed in distilled water, forming a suspension with a concentration of 1.5 M NaOH. The reaction slurry was sonicated for 15 min followed by aging at ambient conditions. Crystallization of the resulting aluminosilicate hydrogel on seeds was carried out hydrothermally at 90 °C for 4 h. The seeds were fine particles of unreacted FA components, mainly metal oxides. The final slurry was filtered, and the synthesized solid product was washed repeatedly with distilled water to a residual pH = 8 and dried at 105 °C. A powdered sample of zeolite was subjected to wet ball milling with a PULVERISETTE 23 Mini-Ball Mill (FRITSCH) with 50 osc/min for three different durations as follows: 60, 120, and 540 s. For wet milling, colloidal solutions were prepared from 0.08 g of Na-X zeolites in 3 mL of distilled water.

The morphology and structure of both milled and not-milled zeolite powders were characterized by Philips 515 scanning electron microscope (SEM) (Amsterdam, Netherlands) and JEOL JEM 2100 transmission electron microscope (TEM) (Tokyo, Japan). The samples for TEM analysis were prepared by dropping a microquantity of the zeolite suspensions on standard copper grids and subsequent drying for 24 h at ambient conditions. The textural properties of all samples were studied by the N_2_-physisorption technique, and the experimental data were subsequently processed by standardized mathematical models. The samples were preliminary degassed at 260 °C for 7 h in a FlowPrep 060 set-up (Micromeritics Instrument Corp., Norcross, GA, USA), cooled down to ambient temperature under the continuous helium flow, and evacuated for one hour with a rate of 0.67 Pa/s. The sorption analyses were performed with N_2_ with a purity of 4 N at cryogenic temperature provided by liquid nitrogen using a volumetric gas analyzer TriStar II 3020 (Micromeritics Instrument Corp., Norcross, GA, USA). The equilibrium adsorption/desorption isotherms were measured at 92 points in the pressure range p/p_0_ = 0.001–0.950, where p_0_ is the N_2_ saturation pressure. The specific surface area (S_BET_) in m^2^/g was calculated by the Brunauer–Emmett–Teller (BET) model applied in the monolayer adsorption pressure range. The surface area (S_micro_, m^2^/g) and the volume (V_micro_, cm^3^/g) provided by micropores and the micopore size (d_micro_, Å) were calculated from the experimental adsorption isotherms by the t-plot model. The mesopore size distribution, the average mesopore diameter (d_meso_, Å), and the mesopore volume (V_meso_, cm^3^/g) were determined by applying the Barrett–Joyner–Halenda (BJH) model to the desorption branch of the isotherms.

The change in the size of the Na-X zeolites before and after wet-milling was studied by dynamic light scattering (DLS, Zetasizer Nano ZS, Malvern Panalytical Ltd, Malvern, United Kingdom) measurements of particles’ hydrodynamic diameter.

Niobium sol was prepared by the sonocatalytic method using NbCl_5_ (99%, Aldrich) as a precursor, according to the recipe described in details in [26]. Briefly, 0.400 g NbCl_5_ (Sigma-Aldrich, St. Louis, Missouri, USA) was added to 8.3 mL ethanol (98%) and 0.17 mL distilled water and subjected to sonication for 30 min. The solution was aged for 24 h at ambient conditions prior to spin coating. Thus, a transparent and stable Nb sol was obtained easily and without additives. Then, solutions with volume concentrations of 1% and 5% of zeolites in niobium sol were prepared as follows: 0.002 mL and 0.01 mL zeolites from the milled water solution with concentration of 2.7% were mixed with 0.030 mL ethanol and added to 0.198 mL and 0.190 mL Nb sol, respectively. For comparison, a thin film of pure Nb_2_O_5_ was deposited using the same sol without the addition of zeolites.

For the deposition of thin Nb_2_O_5_-Na-X films, a Laurell WS-650MZ-23NPPB spin coater (North Wales, Pennsylvania, USA) was used. The spin-coating process was performed by dropping 0.3 mL of the sol/zeolite solutions onto pre-cleaned Si substrates in a step-wise manner: first, the substrate was rotated at 2500 rpm for 1.5 s; then, in the second stage, the rotations and duration were increased to 4000 rpm and 60 s, respectively. In both steps, the acceleration speed was 2500 rpm/s.

The composite films were post annealed in air at 320 °C for 30 min and then reflectance spectra *R* of the films were measured at normal light incidence by UV/VIS/NIR spectrophotometer Cary 05E (Agilent, Santa Clara, CA, USA). Optical constants (refractive index *n* and extinction coefficient *k*) and thickness *d* of the thin films were determined from measured *R* spectra of the layers with an accuracy of 0.01, 0.005, and 1 nm in *n*, *k*, and *d*, respectively, using a previously developed calculating procedure described elsewhere [26]. Rms roughness of the films was measured using a 3D optical profiler (Zeta-20, Zeta Instruments, Milpitas, CA, USA).

**Figure 1:**
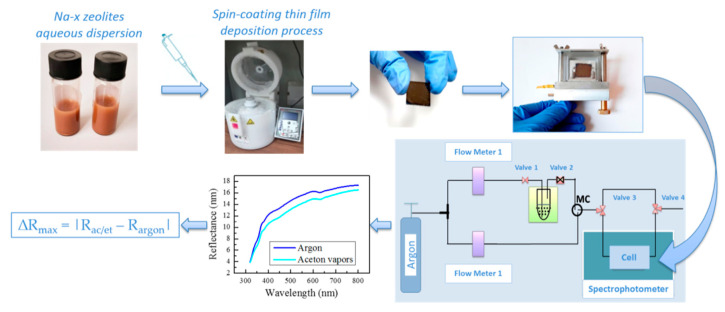
Schematic structure of the process of deposition and testing the sensing properties of the films toward acetone or ethanol vapors.

In order to study the sensing properties of the films, they were placed in a cell that was filled in a cycling manner with argon and acetone/ethanol vapors depending on the exact stage of the measurement [24]. The cell was placed in the spectrophotometer compartment and reflectance spectra were measured successively in argon and VOCs’ vapors environment (Figure 1). It is obvious that all measurements were performed in one and the same spot, thus eliminating the possible difference in spectra that could arise from different spots. This assures that any observed change in reflectance is solely due to exposure to the studied analyte. The sensing abilities of the films were estimated by calculating the maximum change ∆*R*_max_ (%) between spectra taken in argon and acetone/ethanol vapors—∆*R*_max_ = |*R*_ac/et_ − *R*_argon_| (Figure 1). The experimental sequence of the film deposition and the study of their sensing properties when exposed to VOCs are presented in Figure 1.

## 3. Results and Discussion

### 3.1. Zeolites’ Synthesis and Surface Studies

In our previous study, the synthesized material from raw FA was characterized as a zeolite of Na-X type [25]. The phase identification was provided by XRD analysis and confirmed by the SEM images [25]. Intensive diffraction reflexes typical of the Faujasite framework have been found on the experimental diffractogram of the sample [25]. The conducted XRD study of milled samples (not shown here) has confirmed that there is no change of crystal structure due to wet milling. Only a small decrease in diffraction reflexes was obtained after milling. SEM images revealed hexaoctahedral crystallites typical for zeolite Na-X with submicron sizes obtained as a result of the ultrasonic treatment [25]. The iron oxides that are usually presented in the raw FA are transferred into the zeolite product as finely and uniformly dispersed ferric ions and Fe^2+^/^3+^–oxide entities, as confirmed by the Mössbauer studies [25]. The nanocrystalline morphology along with the uniform distribution of the iron components in the zeolite matrix demonstrated the advantages of the ultrasonic-assisted double-stage fusion-hydrothermal synthesis. In this sample, a 94% degree of zeolitization of the raw coal ash was determined by the XRD deconvolution.

**Figure 2:**
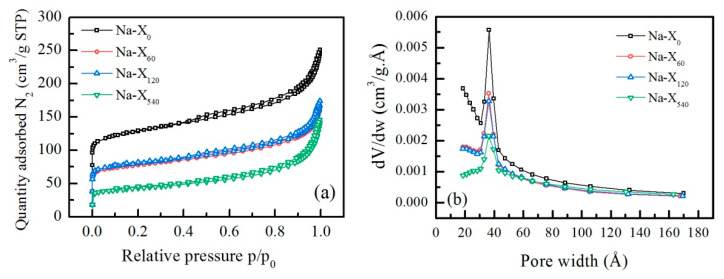
Experimental N_2_ adsorption/desorption isotherms (**a**) and BJH plot of pore size distribution (**b**) of parent Na-X zeolites and zeolites after milling for 60, 120, and 540 s.

The obtained experimental N_2_-adsorption/desorption isotherms of the parent and milled zeolite samples are plotted in Figure 2a. All isotherms could be attributed to Type IV according to the International Union of Pure and Applied Chemistry (IUPAC) classification, which is typical for solids with a mixed micro-mesoporous structure [27].

The fast adsorption at low p/p_0_ is related to the filling in micropores. A comparative examination of the experimental isotherms showed the most intensive adsorption at low pressures in the parent Na-X zeolite, while the amount of the adsorbed N_2_ decreases in the milled samples. For Na-X_60_ and Na-X_120_, the isotherms almost overlap, and the prolongation of the milling time led to the lowest adsorption in the low pressure range for Na-X_540_. The monolayer adsorption is established up to p/p_0_ = 0.05 for all samples, where the linear multipoint BET model was applied in five points with the highest correlation (up to 0.999999). Increasing the pressure, the adsorption in the studied samples continues by filling of mesopores and formation of a multilayer up to p/p_0_ = 0.9. The intensive adsorption in the highest pressure region up to p/p_0_ = 0.995 is indicative of the presence of macropores and filling into the inter-crystal free volume. The desorption and adsorption branches of the isotherms are slightly displaced, describing the hysteresis loops closed at p/p_0_ = 0.35 for all samples. The displacement in the processes of adsorption and desorption relative to the pressure is related to capillary condensation in the mesopores. The experimental desorption data were used as input data in the BJH model to determine the pore size distribution. The plot of the free pore volume distribution (dV/dw,cm^3^/g.Å) versus the pore width is presented in Figure 2b. The results confirm the observation from the N_2_-adsorption/desorption experiments that the non-milled Na-X zeolite possesses the highest free volume indicated by an intensive peak at a pore width range of 35–45 Å, and a trend of increasing the pore volume in the direction of the micropore sizes. The main part of the free volume at all samples is provided by pores with a width of 38Å, as the BJH plots of Na-X_60_ and Na-X_120_ almost overlap but the intensity of the peaks determining the pore volume is reduced as compared with Na-X_0_. The sample Na-X_540_ possesses drastically reduced free pore volume, which shows that the excessively prolonged milling time leads to destruction of the zeolite framework.

In Table 1, the surface characteristics of the studied samples calculated from the experimental isotherms by standardized models are summarized: specific surface area (S_BET_, m^2^/g); external surface (S_external_, m^2^/g); surface described by micropores (S_micro_, m^2^/g); total internal volume (V_total_, cm^3^/g); the volume part determined by micro- (V_micro_, cm^3^/g) and mesopores (V_meso_, cm^3^/g); and average micro- (d_micro_, Å) and mesopore (d_meso_, Å) sizes of initial Na-X zeolite (Na-X_0_) and zeolites after milling for 60 (Na-X_60_), 120 (Na-X_120_), and 540 (Na-X_540_) seconds.

As can be seen, the specific S_BET_ surface of zeolites decreases after the milling process and the reduction is most pronounced for the sample milled for 540 s, in which case S_BET_ = 158.8 m^2^/g is almost twice less than that of zeolites milled for 60 or 120 s. For the samples milled for 60 and 120 s, the difference in the specific surfaces is within the experimental error and the results are identical—around 300 m^2^/g. Despite the lower surface area at Na-X_60_ and Na-X_120_ as compared with the initial Na-X_0_ zeolite, the S_BET_ value of 300 m^2^/g is sufficient for processes taking part on the solid surface. After the milling, the share of micro and mesopores decreases, and the external surface is reduced owing to the particle coalescence. However, the average mesopore width is increased. The effect of milling on the surface parameters is most pronounced in Na-X_540_. It is well known that zeolite Na-X crystallizes as a metastable phase [28]; therefore, prolonged mechanical treatment could cause recrystallization of Na-X in a thermodynamically more stable phase. As Na-X is a zeolite phase with extremely high specific surface area and porosity, its recrystallization into another zeolite phase will lead to deterioration of the surface characteristics of the material. This could also be the probable reason for the observed noticeable increase in the size of the mesopores in the Na-X_540_ sample. However, as already mentioned, the XRD patterns of zeolites are similar. This leads to the conclusion that, if the recrystallization takes place, it is very weak and not detectable by X-ray diffraction.

### 3.2. Zeolites Size

**Figure 3:**
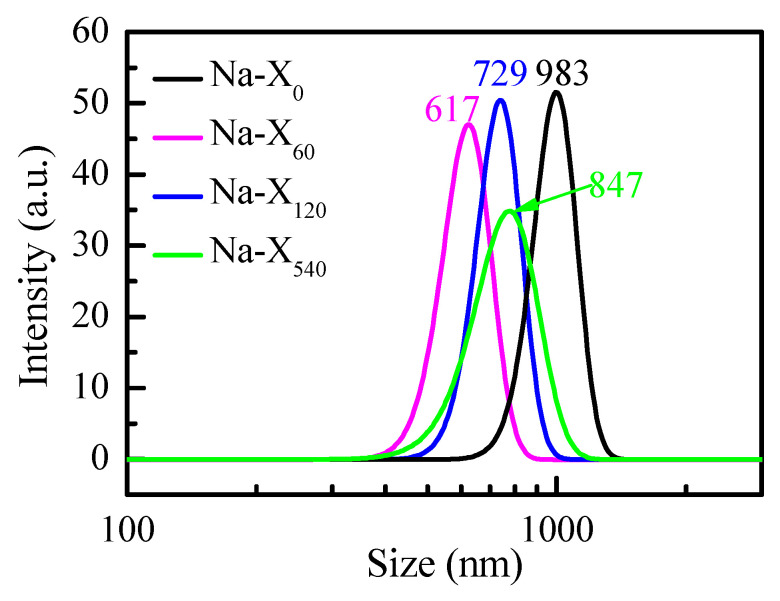
DLS curves for colloidal aqueous solutions of initial Na-X_0_ zeolites and zeolites after milling for 60, 120, and 540 s.

The influence of the milling on the size of the Na-X zeolites was studied by measuring the hydrodynamic diameter of the particles using dynamic light scattering (DLS) (Figure 3). From our previous studies, we know that not milled zeolites with average size around 1 µm impair the optical quality of the composite thin films of Nb_2_O_5_ matrix doped with zeolites. Whereas, in this case too, the initial zeolites Na-X_0_ are of a similar size; therefore, with wet milling, we aim at decreasing the size of the particles and to obtain transparent films with smooth surfaces. This will assure negligible scattering that is vital when optical sensing is considered. As seen from Figure 3, after 60 s of wet milling, the initial size of the zeolites decreases from 983 nm to 617 nm. It is interesting to note that an additional 60 s of milling leads to a slight increase in zeolites’ size from 617 nm to 729 nm. The longest milling with duration of 540 s does not substantially influence the particles size, but significantly changes their size distribution. The width of DLS curve for 540 s increases, thus indicating the presence of particles with various diameters ranging from 350 nm to 1500 nm.

**Figure 4:**
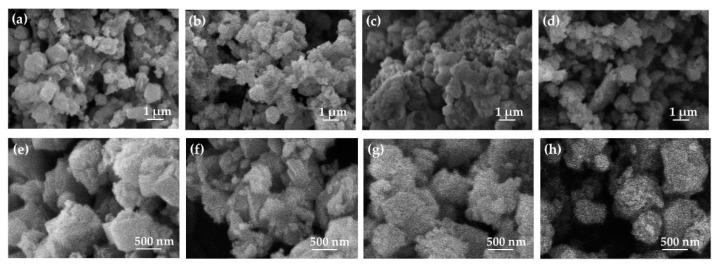
SEM micrographs of powders from initial Na-X_0_ zeolites (**a**,**e**) and zeolites after milling for 60 (**b**,**f**), 120 (**c**,**g**), and 540 (**d**,**h**) seconds at magnifications of 5000^×^ (**a**–**d**) and 20,000^×^ (**e**–**h**).

SEM micrographs of powders from initial Na-X_0_ zeolites and zeolites after milling for 60, 120, and 540 s, of Na-X_60,_ Na-X_120_, and Na-X_540,_ respectively, are presented in Figure 4 in two magnifications. Na-X_0_ is composed of individual hexaoctahedral crystallites (Figure 4a,e). The wet milling of 60 and 120 s results in finer morphology of closely packed nanocrystals, which reduces the free pore volume (Figure 4b,c,f,g). The prolonged milling of Na-X_540_ leads to the particle aggregation and formation of agglomerates, which explains the drastic reduction in the specific surface area and the internal free volume. Simultaneously, the prolonged milling results in widening of the particles’ size distribution, already noticed from DLS curves and confirmed by the SEM pictures presented in Figure 4d,h, where particles with diverse sizes are seen.

**Figure 5:**
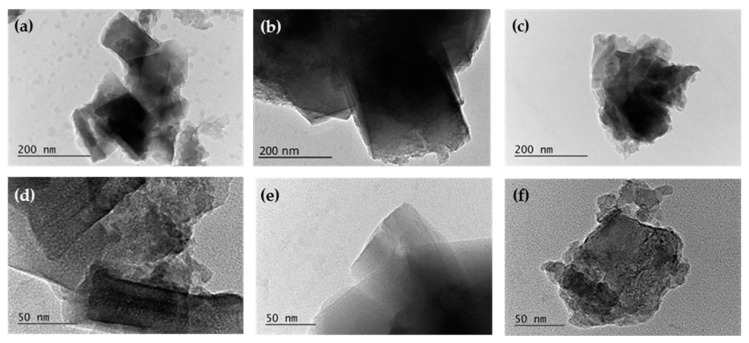
TEM images of non-milled NaX_0_ (**a**,**d**), short-milled Na-X_60_ (**b**,**e**), and long-milled Na-X_540_ (**c**,**f**) zeolite samples.

The morphology of parent and milled zeolites was also investigated using transmission electron microscopy (TEM) at different magnifications. Typical TEM images of Na-X_0_, Na-X_60_, and Na-X_540_ are presented in Figure 5.

In the initial sample, the hexaoctahedral crystallites typical of the Na-X phase are clearly observed, which confirms the formation of a well-organized zeolite structure (Figure 5a,d). Irregularly shaped crystals are also presented, indicating that the zeolites synthesized from by-products are characterized with more structural imperfections than the zeolites derived from pure starting materials. The structural irregularity of these materials may be an advantage in processes related to surface phenomena, as they lead to a stronger surface unsaturation. This is confirmed by the experimentally established high catalytic and adsorption ability of zeolites from FA, comparable to that of their pure synthetic analogues despite their lower specific surface area [25]. At higher magnifications, the fingerprints of the crystalline phase are clearly visible. TEM images of the milled for the short time sample Na-X_60_ show preservation of the typical Na-X crystal morphology (Figure 5b,e). The morphology of Na-X_120_ zeolite is similar, which is why the images are not presented. TEM analysis confirms the observations from surface studies and SEM pictures that prolonged milling of zeolite Na-X_540_ leads to the destruction of crystallites and probable recrystallization of the zeolite phase (Figure 5c,f).

### 3.3. Optical Properties

**Figure 6:**
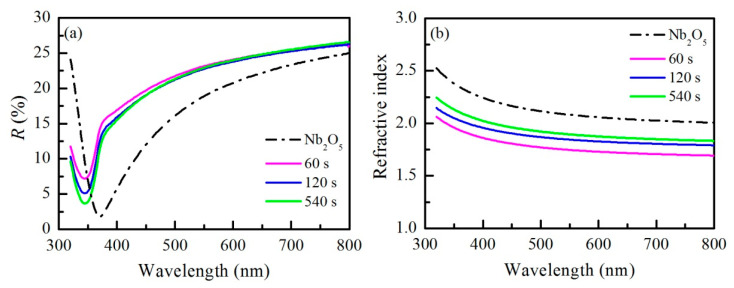
Reflectance spectra (**a**) and dispersion curves of the refractive index (**b**) of thin Nb_2_O_5_ films and composite films of Nb_2_O_5_ doped with 5% Na-X zeolites milled for 60 s, 120 s, and 540 s.

Our recent investigations of Nb_2_O_5_ films doped with zeolites synthesized by long-term alkaline atmospheric conversion of FA have shown that the films’ response towards liquid acetone substantially improves when milled zeolite particles are used as dopants as compared with untreated zeolites [14]. Based on the results of our measurements and data on particle sizes, optical and sensing characterization was focused on thin films doped with milled zeolites. Therefore, Nb_2_O_5_ composite thin films with two different levels of doping with Na-X zeolites (1% and 5%) were deposited on silicon substrates. Pure Nb_2_O_5_ film with no addition of zeolites was also prepared in order to make a comparison with doped films. Afterwards, the reflectance spectra *R* of the films were measured (Figure 6a) at normal light incidence and refractive index *n*, extinction coefficient *k*, and thickness *d* were determined from spectra (Figure 6b and Table 2).

It is seen from Figure 6a that the shapes of the spectra of pure and doped thin Nb_2_O_5_ matrix are similar, but a blue shift takes place when zeolites are introduced into the matrix. The observed shift toward short wavelengths indicates a reduction in optical thickness (refractive index multiplied by the thickness of the film) and could be explained by the decrease in the effective refractive index of the Nb_2_O_5_/Na-X composite films due to addition in the matrix of a medium with low refractive index (Na-X zeolites). We have already shown that zeolites have a much lower refractive index than metal oxides [23]. Further, the measured spectra are used for calculation of the refractive index, *n*; extinction coefficient, *k*; and film thickness, *d*. Calculated dispersion curves of refractive index are shown in Figure 6b. All curves exhibit normal dispersion (*n* decreases with wavelength) that could be expected considering the excellent transparency of the films; the extinction coefficient is less than 0.02 (Table 2). As expected, the refractive index of composite films is lower as compared with the oxide matrix. The reduction in the refractive index depends on the concentration of zeolites and is stronger for highly doped samples. It is seen from Table 2 that, for the composite with 5% Na-X zeolites, the refractive index increases with the duration of milling, while a minimum is observed for the 1% doped composite when Na-X zeolites milled for 120 s are used as dopants. As mentioned above, the introduction of zeolites in the oxide matrix leads to the generation of free volume that finally results in a decrease in the refractive index of composite films as compared with the oxide matrix.

There are two factors that determine the free volume in the films: the intrinsic porosity of the zeolites and the interparticle spaces. The interplay between them governs the porosity of the composite films. Thus, if we assume that the interparticle spaces in the films do not depend on the zeolite size, then the lowest refractive index is expected for films doped with Na-X_120_. The reason is that Na-X_120_ zeolite exhibits the highest pore volume and specific surface area (Table 1), i.e., it has the highest intrinsic porosity. This is exactly what happens for 1% doped films; the refractive index has a minimum for 120 s (Table 2). However, this trend is not observed for heavily doped samples where the refractive index increases with the milling time. When zeolites are introduced in a higher concentration (Na-X-5%), it is very likely that some agglomeration will take place and the zeolite will be dispersed in the matrix less homogenously. Owing to the formation of clusters of several particles, the free volume generated from interparticle spaces decreases, leading to the reduction in overall porosity and an increase in the refractive index. The impact of clustering on refractive index is enhanced with prolonged milling because the size of particles increases, thus further decreasing the overall porosity due to interparticle spaces.

The rms surface roughness of the films was measured and the values are displayed in Table 2. Pure Nb_2_O_5_ films and films doped with 1% Na-X zeolites have similar roughness, while the addition of 5% zeolites leads to a slight increase. The milling duration does not influence the film roughness.

### 3.4. Sensing Experiments

**Figure 7:**
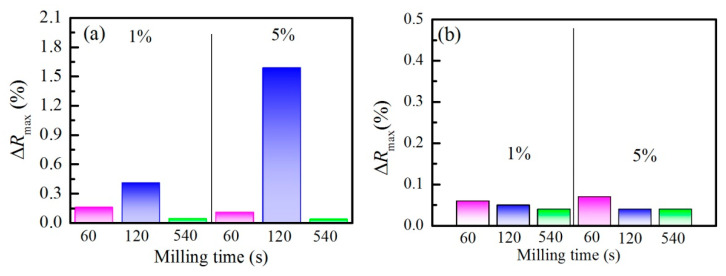
Reflectance change ∆*R*_max_ = |*R*_ac/et_ − *R*_argon_| for Nb_2_O_5_-based composite films doped with Na-X fly ash zeolites with different concentrations (denoted on each plot) milled for 60 s (magenta bars), 120 s (blue bars), and 540 s (green bars) and exposed to acetone (**a**) and ethanol (**b**) vapors.

VOCs’ vapors sensing experiments were conducted using a homemade bubbler system for the generation of acetone and ethanol vapors from liquid. Samples were placed in a cell where the atmosphere can be controlled and changed from argon to ethanol or acetone vapors. In order to evaluate the sensitivity and selectivity of the thin films, reflectance spectra were measured before and during exposure to acetone or ethanol vapors. Calculated changes in reflectance ∆*R*_max_ = |*R*_ac/et_ − *R*_argon_| induced as a result of exposure to acetone and ethanol vapors are shown in Figure 7a,b, respectively. The exposure of the composite film to acetone/ethanol results in vapor condensation in the zeolite pores and replacement of the air inside with acetone/ethanol with a higher refractive index. As a result, the effective refractive index of the film increases, thus leading to a change in the reflectance spectra and increase in ∆*R*_max_.

For acetone vapors, the same dependence of the reflectance change ∆*R*_max_ on the milling time is clearly observed for both zeolite concentrations. The greatest change is obtained for films doped with zeolites milled for 120 s, and the smallest for 540 s (Figure 7a). There is a very weak concentration dependence of ∆*R*_max_ when zeolites are milled for 60 s and 540 s. However, when the milling time is 120 s, there is a strong concentration dependence of reflectance change; that is, ∆*R*_max_ for heavily doped sample is 1.6%, thus being almost four times higher than the response of the lightly doped one, which is 0.4%. We may conclude that there are optimal conditions under which the reaction to acetone vapors is the greatest, depending on the concentration of zeolites, their size, and the milling time. It is seen from Table 1 that the pore volumes V_total_, V_micro_, and V_meso_ as well as the specific surface area S_BET_ and the area described by micro pores (S_micro_) are the highest for zeolites milled for 120 s as compared with those milled for 60 s and 540 s. The biggest pore volumes along with the highest specific surface areas of Na-X_120_ zeolites facilitate the vapor condensation and are the possible reason for the enhanced sensitivity observed for the Nb_2_O_5_/Na-X_120_ sample. The deterioration of the sensitivity for a longer milling time (540 s) is most probably due to the decreased pore volumes and specific surface areas of Na-X_540_ samples (Table 1). Moreover, the data from optical characterization confirm that the overall porosity of this sample decreases. It is interesting to note that the pore volumes and specific surface areas of Na-X_60_ zeolites are close to the values of Na-X_120_, but the response of the Nb_2_O_5_/Na-X_60_ films is lower as compared with the films consisting of Na-X_120_ zeolites, and this is mostly pronounced in the case of heavily doped samples. Moreover, in this case, the refractive index on Nb_2_O_5_/Na-X_60_ is lower as compared with Nb_2_O_5_/Na-X_120_, which means more free volume exists in the sample. It seems that, although the overall porosity of the Nb_2_O_5_/Na-X_60_ sample is higher, not all pores are accessible for the vapors, i.e., the dominating porosity in this sample is of the closed porosity type.

In order to study the selectivity, similar experiments were performed, but with ethanol vapors. The results for ∆*R*_max_ are summarized in Figure 7b. It is seen that there is a significant decrease in films’ response to ethanol vapors as compared with acetone. The maximum value for ∆*R*_max_ achieved is 0.07% which is more than 20 times smaller than the response towards acetone vapors. This result clearly indicates the selectivity of Nb_2_O_5_/Na-X composite thin films toward acetone. As is well known, acetone has a rather extended electron charge cloud, as evidenced by a molecular diameter of 6.2 Å, while the diameter of ethanol molecules is 4.4 Å. As seen from Table 1, the size of the pores is sufficient for both analyte molecules to penetrate. Favorable formation of reactive complexes between the ketones and the Brønsted acid sites into the zeolite framework via the proton exchange mechanism is widely used as a precursor stage for many organic reactions [29]. However, ethanol could be also attached to the Brønsted acid sites, forming surface ethoxy species [30]. Thus, the most likely reason for greater acetone reaction is the higher reactivity of the acetone molecules owing to their stronger polarizability [31].

**Figure 8:**
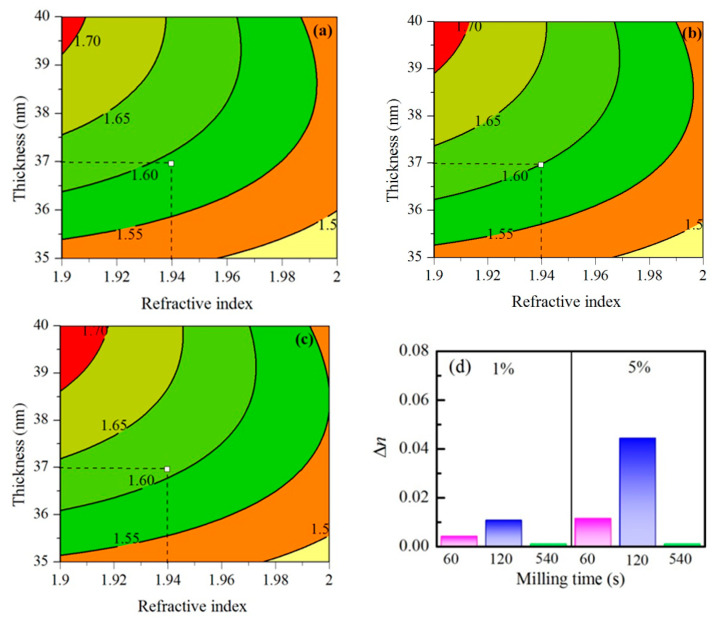
Contour plots (**a**–**c**) of calculated values of reflectance change Δ*R*_max_ (%) for Nb_2_O_5_ thin film doped with 5% of Na-X_120_ as a function of refractive index and thickness. The calculations were performed at a wavelength of 410 nm and Δ*n* = 0.0442 (**a**), Δ*n* = 0.0444 (**b**), and Δn = 0.0446 (**c**). The white square with coordinates *n* = 1.94 and *d* = 37 nm presents the refractive index (at wavelength of 410 nm) and thickness of the film, respectively; (**d**) calculated values of Δ*n* using the contour plot approach.

As mentioned above, the observed reflectance change when films are exposed to vapors is due to the increase of effective refractive index (Δ*n*) of the composite films, which is a consequence of the condensation of vapors in the zeolite pores. For the calculation of Δ*n* of each sample, a recently developed approach was used [32]. It is based on fitting the discrepancies between calculated and measured values of Δ*R*_max_. In this regard, a series of calculations of Δ*R*_max_ in the *n*-*d* plane were performed at fixed wavelength and different values of Δ*n*. The latter was varied until the measured and calculated values of Δ*R*_max_ coincided at the point with coordinates (*n*,*d*) (*n*, *d*—refractive index and thickness of the film, respectively). Figure 8 shows typical contour plots of calculated values of reflectance change Δ*R*_max_ for Nb_2_O_5_/Na-X_120_ composite film as a function of the refractive index and thickness at a wavelength of 410 nm for three different values of Δ*n*: 0.0442, 0.0444, and 0.0446. The particular value of wavelength for this sample is selected as a wavelength where Δ*R* reaches its maximal value of 1.6%. It is seen from Figure 8 that, for the Nb_2_O_5_/Na-X_120_ composite film with *d* = 37 nm and *n* = 1.92 (at 410 nm), a match between measured and calculated Δ*R*_max_ is obtained for Δ*n* = 0.0444 (Figure 8b). When Δ*n* is smaller (Figure 8a, Δ*n* = 0.0442) or higher (Figure 8c, Δ*n* = 0.0446), the point that represents the film deviates from the 1.6% contour line. This means that there is no a match between the measured and calculated Δ*R*_max_ values.

The calculated values of Δ*n* using the contour plot approach are summarized in Figure 8d and Table 2. The observed dependences are similar to the dependences of Δ*R*_max_ presented in Figure 7a. It is confirmed that composite films doped with 5% Na-X_120_ zeolites exhibit the greatest absorption ability towards acetone vapors. Owing to condensation of vapors in the pores, the effective refractive index increases with 0.0444. It is interesting to note that these values are very close to Δ*n* values of 0.048 obtained for mesoporous Nb_2_O_5_ thin films exposed to acetone vapors reported in [33]. The porosity of the latter is about 50% and was generated using a commercial organic template [33].

**Figure 9:**
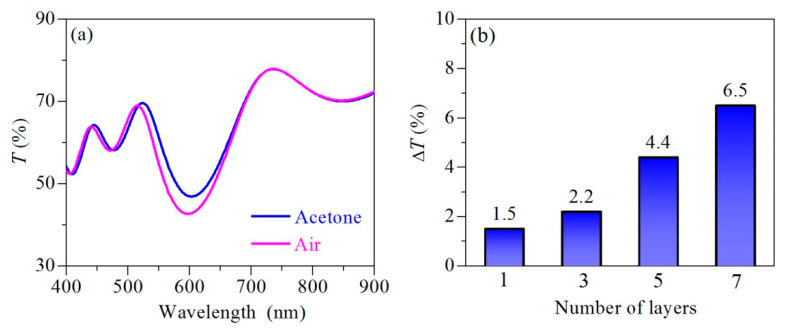
(**a**) Calculated transmittance spectra *T* in air and acetone vapors of a computer-modeled seven-layer Bragg stack consisting of undoped Nb_2_O_5_ matrix and matrix doped with 5% Na-X_120_ zeolites; (**b**) calculated change in transmittance ∆*T* = |*T*_ac_ − *T*_argon_| due to acetone vapors exposure as a function of the number of the layers in the stack.

One possible way to enhance the sensitivity of a single film is to incorporate it in a multilayer structure. The latter could be, for example, a quarter-wavelength stack referred to as a Bragg stack. It consists of alternating films with a low and high refractive index. Because of the quarter-wavelength optical thickness of the layers, all multiple reflected waves are in phase and interfere constructively, generating a band of high reflectance. On the contrary, all multiple transmitted waves interfere destructively, causing the appearance of a low transmittance band (Figure 9a). The spectral position of the bands depends on the optical thickness and a shift is observed if layers of the refractive index and/or thicknesses change (Figure 9a).

In order to explore the possibility of utilization of Nb_2_O_5_/NaX_120_ film as a building block for a vapor-responsive Bragg stack, we calculated the transmittance spectra in air and vapors of acetone of a theoretical Bragg stack with three, five, and seven layers (Figure 9a), and compared ∆*T* = |*T*_ac_ − *T*_argon_| with the value for a single film (Figure 9b). The modeled stacks comprise undoped Nb_2_O_5_ matrix as a layer with a high refractive index (*n*_H_) and Nb_2_O_5_ doped with 5% Na-X_120_ zeolites as a low refractive index layer (*n*_L_). The exposure to acetone vapors (the acetone spectrum in Figure 9a) is modelled by increasing of *n*_L_ with ∆*n* = 0.0444 (this value is the change in the refractive index of Nb_2_O_5_/NaX_120_ film calculated using the contour plot approach described above). A gradual increase in ∆*T* with the number of the layer in the stack is obtained and an enhancement with a factor of more than 4 is achieved when a seven-layer stack is considered (Figure 9b). The benefit of the multilayer structure is very well distinguished, especially if one compares ∆*T* values for single-layer and three-layers stacks. The latter consists of an Nb_2_O_5_/NaX_120_ composite layer stacked between two Nb_2_O_5_ films; therefore, the sensitive medium is the same for single layer and three-layer stacks. However, ∆*T* increases from 1.5% to 2.2%, i.e., a 50% increase in ∆T is obtained if the sensitive Nb_2_O_5_/NaX_120_ composite layer is incorporated between two Nb_2_O_5_ layers (Figure 9b).

**Figure 10:**
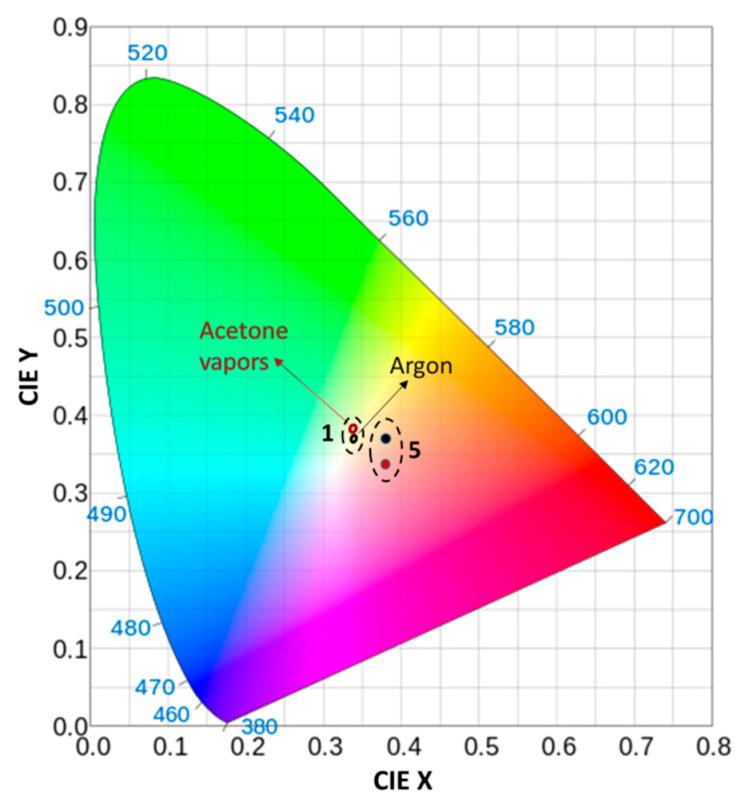
Calculated CIE coordinates for Nb_2_O_5_ film with Na-X_120_ zeolites with a concentration of 5% (label 1) and a computer-modelled five-layer Bragg stack comprising Nb_2_O_5_ as a high refractive index layer and Nb_2_O_5_/Na-X_120_ as a low refractive index layer (label 5) exposed to argon (black circles) and acetone vapors (red circles).

The next step in our investigation was to study the possibility of using the composite films for color sensing. Color sensing is a simple method for vapors’ detection and it is based on perceptual color change due to the presence of analyte. If a transparent film is deposited on an opaque substrate (silicon wafer, in our case), it exhibits a certain color depending on its optical thickness (the physical thickness multiplied by the refractive index). If thin film’s optical thickness changes due to the adsorption of vapors, the reflectance spectrum of the film will shift. As a result, the color of the film will change with the strength of the reflectance shift. The possibility of monitoring the color in response to different analytes is studied by calculating color coordinates CIE X and CIE Y of the thin films doped with zeolites prior to and during exposure to acetone vapors. The calculated color coordinates CIE X and CIE Y of a single Nb_2_O_5/_Na-X_120_ film and theoretically modeled five-layer Bragg stack comprising Nb_2_O_5_ as a high refractive index layer and Nb_2_O_5_/Na-X_120_ as a low refractive index layer are plotted in Figure 10. In the case of a single film, reflectance spectra prior to and during analyte exposure are used for calculation, while for Bragg stack, the modelled transmittance spectra are used.

Each point on the CIE plot represents the color of the sample prior to and during exposure to acetone vapors. For single Nb_2_O_5_/Na-X_120_ deposited on silicon substrate, the two points are close to each other, which means the change in the color due to exposure to the analyte is not so distinct. However, the two points do not overlap, i.e., they are separated in the color space, thus enabling color detection of acetone vapors. The picture improves substantially if a Bragg stack is implemented for color sensing instead of a single film. It is seen that, in this case, the two points are well separated in the CIE color space, which confirms the possibility of successful application of FA zeolites for color sensing of acetone vapors.

## 4. Conclusions

Zeolite Na-X synthesized by ultrasonic-assisted double-stage fusion-hydrothermal alkaline conversion of coal fly ash are successfully incorporated into the Nb_2_O_5_ thin matrix and the composite films thus obtained are applied as sensitive elements for optical sensing of vapors. The enhancement of the sensitivity toward acetone along with achieving good optical quality of the composite films are realized via wet-milling of the zeolites’ powder for 60, 120, and 540 s prior to film deposition. It is demonstrated that the milling time influences pore volumes, specific surface area, particle size, films’ morphology, and optical and sensing properties of the composite thin films.

In the case of vapor sensing, an optimal milling time of 120 s is obtained. At this milling duration, the highest values of both pore volumes and specific surface areas of Na-X_120_ zeolites are achieved, which facilitate the vapor condensation in the pores, thus enhancing the sensitivity of the Nb_2_O_5_/Na-X_120_ films. A deterioration of the response is observed for the longer milling time (540 s), most probably owing to the decreased pore volumes and specific surface areas of the Na-X_540_ samples. Although the pore volumes and specific surface area of Na-X_60_ and Na-X_120_ zeolites are similar, the Nb_2_O_5_/Na-X_60_ composite films have lower sensitivity as compared with Nb_2_O_5_/Na-X_120_. The possible explanation is the presence of closed porosity in the composite thin film with Na-X_60_ zeolites_._ Very good selectivity toward acetone is obtained; the reflectance change is more than 20 times higher when films are exposed to acetone vapors as compared with exposure to ethanol vapors.

It is demonstrated that composite Nb_2_O_5_/Na-X_120_ films can be utilized for color sensing of acetone vapors. Computer modelling has revealed that a considerable increase in sensitivity can be achieved by the incorporation of sensitive Nb_2_O_5_/Na-X_120_ films into Bragg stacks.

## Figures and Tables

**Table 1:** Surface characteristics of parent and milled coal fly ash zeolite Na-X.

Sample/ Variable	S_BET_, m^2^/g	S_micro_, m^2^/g	S_external,_ m^2^/g	V_total_, cm^3^/g	V_micro_, cm^3^/g	V_meso_, cm^3^/g	d_micro,_ Å	d_meso,_ Å
Na-X_0_	486.3	334.3	166.3	0.307	0.133	0.174	13.9	41.8
Na-X_60_	292.5	197.9	94.5	0.192	0.079	0.113	13.8	43.9
Na-X_120_	305.6	214.0	91.6	0.199	0.085	0.115	13.9	44.9
Na-X_540_	158.8	92.2	66.6	0.139	0.038	0.101	13.6	52.8

**Table 2:** Refractive index *n*, extinction coefficient *k*, thickness *d*, surface rms roughness *S_q_*, and refractive index change ∆*n* upon exposure to acetone vapors of thin films from pure Nb_2_O_5_ and Nb_2_O_5_ doped with Na-X zeolites wet milled for 60, 120, and 540 s.

Sample/ Variable	Pure Nb_2_O_5_	Na-X (1%)/Nb_2_O_5_			Na-X (5%)/Nb_2_O_5_		
		60 s	120 s	540 s	60 s	120 s	540 s
n *	2.06	2.00	1.93	1.98	1.73	1.83	1.87
k *	0.017	0.018	0.018	0.018	0.017	0.017	0.017
*d* (nm)	37	33	34	34	39	37	36
*S_q_* (nm)	4.5 ± 1.2	5 ± 0.3	5.8 ± 1.4	4.3 ± 1	8 ± 2.4	6.2 ± 1.4	6.6 ± 0.9
∆*n*	-	0.42 × 10^−2^	1.08 × 10^−2^	<1 × 10^−3^	1.16 × 10^−2^	4.44 × 10^−2^	1 × 10^−3^

* Values of *n* and *k* are taken at a wavelength of 600 nm.

## Data Availability

Not applicable.
